# Occurrence and Molecular Characteristics of Microsporidia in Captive Red Pandas (*Ailurus fulgens*) in China

**DOI:** 10.3390/ani13111864

**Published:** 2023-06-03

**Authors:** Jinpeng Yang, Yangyang Zeng, Caiwu Li, Songrui Liu, Wanyu Meng, Wenqing Zhang, Ming He, Liqin Wang, Zhili Zuo, Chanjuan Yue, Desheng Li, Guangneng Peng

**Affiliations:** 1 The Key Laboratory of Animal Disease and Human Health of Sichuan Province, College of Veterinary Medicine, Sichuan Agricultural University, Chengdu 611130, China; yangjinpeng@stu.sicau.edu.cn (J.Y.);; 2 China Conservation and Research Center for the Giant Panda, Key Laboratory of State Forestry and Grassland Administration on Conservation Biology of Rare Animals in the Giant Panda National Park, Chengdu 610083, China; 3 Sichuan Key Laboratory of Conservation Biology for Endangered Wildlife, Chengdu Research Base of Giant Panda Breeding, Sichuan Academy of Giant Panda, Chengdu 610081, China; 4 Chengdu Zoo, Chengdu 610051, China

**Keywords:** *Enterocytozoon bieneusi*, *Encephalitozoon cuniculi*, *Encephalitozoon intestinalis*, *Encephalitozoon hellem*, red panda

## Abstract

**Simple Summary:**

Microsporidia are obligatory intracellular fungi that cause infection in a broad variety of eukaryotes. However, there are few epidemiological studies on microsporidia in red pandas in China. Therefore, we conducted an epidemiological survey of microsporidia in red pandas from six zoos in Sichuan Province, China. In 198 fecal samples, *Enterocytozoon bieneusi* was detected in 24 samples (12.1%), while *Encephalitozoon* spp. was detected in 8 fecal samples (4.0%). Further analysis revealed the presence of genotypes of *E. bieneusi* with zoonotic concerns. Interestingly, we also observed that the infection rate of microsporidia was higher in semi-free red pandas with more frequent contact with other animals or humans. This is the first report on the occurrence and genotypes of *E. bieneusi* and *Encephalitozoon* spp. in red pandas in Sichuan Province, China, contributing to our understanding of the worldwide distribution and genetic diversity of this pathogen.

**Abstract:**

*Enterocytozoon bieneusi* and *Encephalitozoon* spp. are microsporidian pathogens with zoonotic potential that pose significant public health concerns. To ascertain the occurrence and genotypes of *E. bieneusi* and *Encephalitozoon* spp., we used nested PCR to amplify the internal transcribed spacer (ITS) gene and DNA sequencing to analyze 198 fecal samples from red pandas from 6 zoos in China. The total rate of microsporidial infection was 15.7% (31/198), with 12.1% (24/198), 1.0% (2/198), 2.0% (4/198) and 1.0% (2/198) for infection rate of *E. bieneusi*, *Encephalitozoon cuniculi*, *Encephalitozoon intestinalis* and *Encephalitozoon hellem*, respectively. One red panda was detected positive for a mixed infection (*E. bieneusi* and *E. intestinalis*). Red pandas living in semi-free conditions are more likely to be infected with microsporidia (χ^2^ = 6.212, df = 1, *p* < 0.05). Three known (SC02, D, and PL2) and one novel (SCR1) genotypes of *E. bieneusi* were found. Three genotypes of *E. bieneusi* (SC02, D, SCR1) were grouped into group 1 with public health importance, while genotype PL2 formed a separate clade associated with group 2. These findings suggest that red pandas may serve as a host reservoir for zoonotic microsporidia, potentially allowing transmission from red pandas to humans and other animals.

## 1. Introduction

Microsporidia are obligate intracellular parasites with more than 1700 species and have been described as opportunistic agents, widely parasitizing arthropods, birds, mammals, and humans [1]. Among the 15 pathogenic microsporidia, *Enterocytozoon bieneusi* and *Encephalitozoon* spp. are recognized as the main causes of microsporidiosis in animals and humans [2]. The fecal-oral route is considered to be the major route of microsporidia transmission, such as ingestion of water and food contaminated with spores. The majority of reports of asymptomatic microsporidial infections occur in immunocompetent individuals. However, when it comes to immunocompromised individuals, such as those with HIV/AIDS, B-cell acute lymphoblastic lymphoma, multiple myeloma, and organ transplant recipients, microsporidia are often seen as the primary pathogen causing their chronic diarrhea [1]. Noteworthily, microsporidial keratoconjunctivitis has been increasingly reported in healthy populations [3].

The spores of microsporidia are so tiny (1–10 μm) that they are difficult to identify by traditional morphological observation, which prompted the detection and genotyping of microsporidia by PCR amplification of the ribosomal RNA (rRNA) internal transcribed spacer (ITS) gene [4,5]. Among these four microsporidia (*E. bieneusi and Encephalitozoon* spp.), *E. bieneusi* is widely prevalent worldwide. Currently, there are over 600 different *E. bieneusi* genotypes, classified into 11 groups based on phylogenetic analyses of the ITS gene [6]. Genotypes belonging to groups 1 and 2 (CD2, CAF1, BEB6, and J) are considered potentially zoonotic, while groups 3–11 are mainly host-specific and non-hazardous [7,8]. A new group of *E. bieneusi* denominated Group 2-like has been proposed previously [9]. Sequence analysis of the ITS gene has so far revealed four genotypes of *Encephalitozoon cuniculi* (I, II, III, and IV) and three genotypes of *Encephalitozoon hellem* (1A, 2, and 3) [10,11]. There is no evidence of intraspecific polymorphisms in the ITS gene of *Encephalitozoon intestinalis* in any study [12,13].

The red panda is considered an endangered species by the International Union for Conservation of Nature, while in China, they are classified as a Category II-protected species under the Wild Animal Protection Law [14]. Over the past few years, epidemiological studies on microsporidia in China have been carried out in both humans and animals, including AIDS patients, livestock, wildlife, and pets [15,16,17,18]. Although *E. bieneusi* infections in red pandas from wildlife parks and wildlife rescue and breeding centers were first reported in China in 2015 [19], the occurrence and genetic characterization of four microsporidian species in red pandas remain poorly studied. *E. bieneusi* genotypes belonging to zoonotic group 2 have been reported in the past in red pandas [19,20], suggesting that red pandas could be a source of zoonotic microsporidia. Previous studies have reported that parasitic infections are currently the most significant threat to the survival of pandas [21]. Therefore, epidemiological surveys of microsporidia in red pandas have an important role in their conservation. The aim of this study was to identify the incidence and genotyping of four microsporidia species in red pandas in Sichuan Province, southwest China.

## 2. Materials and Methods

### 2.1. Ethics Statement

The institutional animal ethics committee at China’s Sichuan Agricultural University gave its approval to this investigation. Every experiment was carried out in conformity with the ethical standards and legal requirements. All stool samples were sampled with the permission of six zoos.

### 2.2. Sample Collection

**Figure 1:**
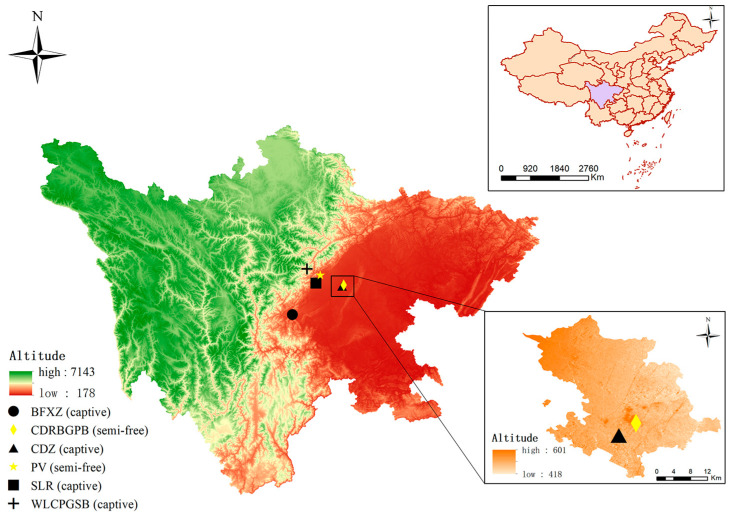
Specific locations for collecting fecal specimens from red pandas in Sichuan province, southwest China. BFXZ: Bifengxia Zoo; CDRBGPB: Chengdu Research Base of Giant Panda Breeding; CDZ: Chengdu Zoo; PV: Panda Valley; SLR: Sanlang Resort; WLCPGSB: Wolong China Panda Garden Shenshuping Base.

In total, 198 stool specimens of red pandas were sampled between July and December 2020 at 6 sites in southwestern China, including Chengdu Research Base of Giant Panda Breeding (CDRBGPB), Panda Valley (PV), Wolong China Panda Garden Shenshuping Base (WLCPGSB), Chengdu Zoo (CDZ), Bifengxia Zoo (BFXZ), and Sanlang Resort (SLR) in Sichuan Province (Figure 1). Of these, 85 fecal samples were collected from 85 captive red pandas, and 113 fecal samples were collected from 113 semi-free red pandas. All red pandas included in the study were in good health and showed no abnormal clinical signs at the time of fecal sampling. Most red pandas were bred in captivity, while a small portion came from wild rescue efforts. The keepers from the six locations were informed of the fecal collection process in advance. Each red panda was sampled only once to collect its feces. All samples were collected with sterile gloves within 24 h after defecation by keepers, immediately transported to the clinic veterinary laboratory of the Sichuan Agricultural University with ice bags, and then stored at −20 °C until DNA isolation. All red pandas were studied in two groups, depending on their range of motion and level of human contact. Group i captive represents the red pandas that are kept in cages and do not contact tourists except for the keepers. The zoo carefully regulates the diet of captive red pandas, sourcing their food exclusively from the market and prohibiting tourists from feeding them. Group ii semi-free has a high level of human contact, as red pandas are free to move around within a certain area and have access to other animals or visitors. Semi-free captive red pandas have close contact with visitors without strict fences or personnel to restrict visitors from entering. Despite the presence of designated areas and fences between different animals, they have the opportunity to venture into other animals’ exclusive zones. Additionally, other animals, such as primates, are known to enter the red panda activity area. They have housing accommodations with unrestricted access. Semi-free red pandas are partially fed by keepers, but they also receive food from visitors.

### 2.3. DNA Isolation and PCR Amplification

The stool sample was suspended in distilled water and mixed thoroughly. The suspension was then filtered through a 250-μm metal mesh filter. The filtrate was transferred to a 50-mL centrifuge tube and centrifuged at 3000 rpm for 5 minutes. The supernatant was removed, and the sediment was used for DNA extraction. Genomic DNA was isolated from around 200 mg of processed feces using the E.Z.N.A.^®^ stool DNA Kit (OMEGA, Biotek Inc., Norcross, GA, USA). Prior to PCR amplification, the isolated DNA was stored at −20 °C. The partial ribosomal ITS region of *E. bieneusi* was amplified using nested PCR to determine the genotypes of *E. bieneusi*, as reported previously [22]. The multi-locus sequence typing (MLST) tool at MS1, MS3, MS4, and MS7 loci was further performed to characterize ITS-positive samples [23]. *Encephalitozoon* spp. were identified by amplification of an ITS rRNA gene of ~300 bp [24]. All primers, cycling conditions, and amplicon sizes are described in Supplementary Table S1. All PCR tests were conducted using a 25 μL reaction mixture containing 12.5 μL of Premix Taq™ (TaKaRa Bio, Otsu, Japan), 8.5 μL of sterilized ddH2O, 1 μL of each primer, and 2 μL of genomic DNA. Both positive and negative controls were used in each PCR run. All PCR reaction products were electrophoresed on a 1.5% agarose gel containing TS-GelRed (Tsingke Biotechnology Co., Ltd., Beijing, China) and visualized under ultraviolet light.

### 2.4. Sequencing and Phylogenetic Analysis

Positive PCR products were delivered to Tsingke Biotechnology Co., Ltd. (Chengdu, China) for bidirectional sequencing using an ABIPRISMTM 3730XL DNA Analyzer. Alignment and correction of bidirectional sequencing results were done using ClustalW (https://www.ebi.ac.uk/Tools/msa/clustalw2/, accessed on 16 February 2022). A comparison of the corrected sequences with reference sequences in GenBank (https://blast.ncbi.nlm.nih.gov, accessed on 16 February 2022) was performed to determine the species and genotypes of microsporidia using the nucleotide Basic Local Alignment Search Tool (BLAST) program. Phylogenetic trees were constructed using neighbor-joining trees based on the evolutionary distances calculated by the Kimura-2-parameter model in the MEGA11 software (https://megasoftware.net, accessed on 17 February 2022). The dependability of the tree was evaluated using the bootstrap method with 1000 repetitions.

### 2.5. Statistics Analyses

To analyze differences in prevalence among regions and activity pattern in this study, the Chi-square test was used with SPSS version 22.0 (IBM Armonk Corp., Armonk, NY, USA). Statistical significance was defined as *p*-values < 0.05.

### 2.6. Nucleotide Sequence Accession Numbers

The ITS sequences of *E. bieneusi* have been deposited in the GenBank database and assigned accession numbers MW880217-MW880236 and MW880238-MW880241. Additionally, the nucleotide sequences of the MS1, MS3, MS4, and MS7 loci of *E. bieneusi* have also been assigned GenBank database accession numbers MW922590-MW922622. The sequences of *Encephalitozoon* spp. have been deposited in GenBank with accession numbers OM731710-OM731713 and OM738338-OM738341.

## 3. Results

### 3.1. Prevalence of Microsporidia in Fecal Samples

The ITS genes of 4 microsporidian species were amplified in 198 fecal specimens, of which 31 (15.7%) red pandas were positive for microsporidia infection. Further, 24 red pandas (12.1%) were detected to be infected with *E. bieneusi*, (Table 1). The overall prevalence of *Encephalitozoon* spp. in red pandas was 4.0% (8/198), with 2.0% (4/198) in *E. intestinalis* and 1.0% (2/198) in both *E. cuniculi* and *E. hellem*. Moreover, one sample was confirmed to be co-infected with *E. bieneusi* and *E. intestinalis*.

Positive samples for microsporidia were detected in all regions, with the prevalence of microsporidia ranging from 0 to 24%, but the differences between regions were not significant (χ^2^ = 7.566, df = 5, *p* > 0.05). More red pandas in Group ii (21.2%) had a higher infection rate with microsporidia compared to Group i (8.2%) (χ^2^ = 6.212, df = 1, *p* < 0.05).

### 3.2. Genotypes of E. bieneusi and Homology Comparison

Four distinct genotypes of *E. bieneusi*, including three known (D, PL2, and SC02) and one new (SCR1) genotype, were identified. The genotypes D, PL2, and SC02 were identical with the reference sequences KY950534, MT497891, and KU852476 from GenBank, respectively. The homology between the new genotype SCR1 and genotype FJL (MK357781) was 98.6% with two single nucleotide polymorphisms (Supplementary Figure S1). Of these, genotype PL2 (75.0%, *n* = 18) was the most prevalent, followed by genotype SCR1 (12.5%, *n* = 3) and SC02 (8.3%, *n* = 2), while genotype D was found in only one sample (Table 1).

Homology comparison of four *E. intestinalis* (OM738338-OM738341) revealed varying degrees of similarity with *E. intestinalis* isolates with 100% query cover value, ranging from 86.21% to 93.29% with isolates reported in immunocompromised patients (KP735194 and KP735187), and 87.30% with an isolate reported in pigeons (AB897501). The *E. cuniculi* detected in red pandas from CDRBGPB (OM731710 and OM731711) showed the highest homology with *E. cuniculi* isolates reported in *Gorilla beringei beringei* in Rwanda (94.85%, KJ577583) with a query cover value of 98%, and *Lagurus lagurus* in Czech Republic (95.53%, KJ469979) with a query cover value of 95.53%, respectively. *E. hellem* from CDRBGPB (OM731712) and CDZ (OM731713) shared homology with *E. hellem* isolates, with similarities of 93.10% (CP075157) and 87.25% (KM459507), respectively. 

### 3.3. Phylogenetic Relationship of E. bieneusi and Encephalitozoon spp.

**Figure 2:**
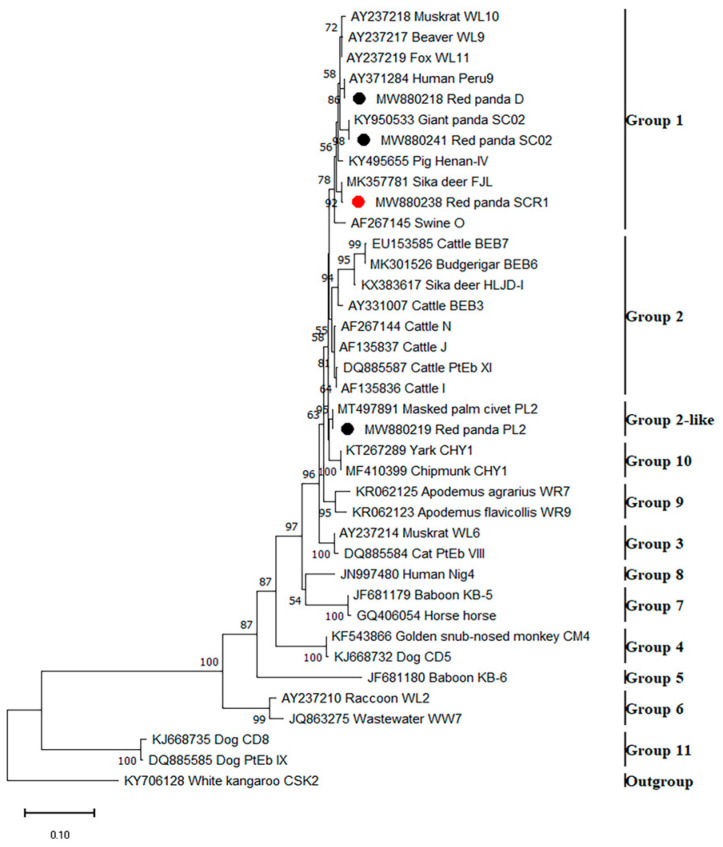
Phylogenetic relationship of *Enterocytozoon bieneusi* genotypes based on ITS sequences by the neighbor-joining method. The percentage bootstrapping values on the branches are based on 1000 repetitions, and values of more than 50% are displayed in the tree. The black and red circles, respectively, represent the known and new *E. bieneusi* genotypes found in this study.

**Figure 3:**
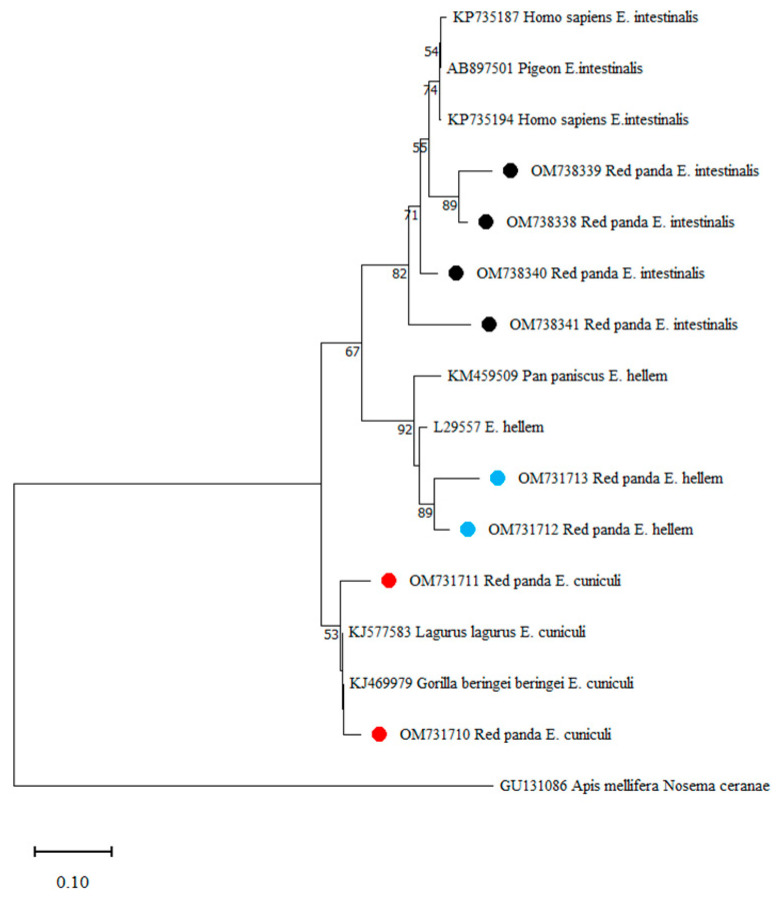
Phylogenetic relationship of *Encephalitozoon* spp. based on ITS sequences by the neighbor joining method. The percentage bootstrapping values on the branches are based on 1000 repetitions, and values more than 50% are displayed in the tree. The black, blue and red circles respectively, represent the *Encephalitozoon intestinalis*, *Encephalitozoon hellem*, *Encephalitozoon cuniculi* found in this study.

The obtained sequences representing the 4 distinct genotypes of *E. bieneusi* were analyzed phylogenetically along with sequences that represent the 11 known groups of *E. bieneusi* (Figure 2). The isolates with three genotypes (SC02, D, SCR1) in this study belonged to group 1. Genotype PL2 formed a separate branch known as the group 2-like, which is associated with group 2. The phylogenetic analysis of the obtained sequences of *Encephalitozoon* spp. was also performed (Figure 3).

### 3.4. Multi-Locus Sequence Typing of E. bieneusi

To improve the reliability of the characterization of *E. bieneusi* subtypes isolated from red pandas, the 24 ITS-positive samples were analyzed more thoroughly by MLST at 4 loci (MS1, MS3, MS4, and MS7). At least 62.5% of samples were successfully genotyped at one locus. Further, 25.0% (6/24), 45.8% (11/24), 16.7% (4/24), and 50.0% (12/24) of ITS-positive samples were successfully amplified at four loci (Table 2). In total, three haplotypes at all four loci and three different multi-locus genotypes (MLGs) were identified. All three MLGs (MLG1, MLG2, and MLG3) were discovered in *E. bieneusi* with genotype PL2.

## 4. Discussion

Although waterborne transmission is thought to be the primary route of microsporidia infection in humans and animals, the possible routes and sources of transmission are still not entirely defined. In recent years, more studies have been done on animal microsporidiosis to determine the source of the disease and evaluate zoonotic risk, but less on the red panda as a potential host. Herein, we performed the initial epidemiological survey of *E. bieneusi* and *Encephalitozoon* spp. and demonstrated the presence and genotypic characteristics of microsporidian species in red pandas in Sichuan Province, Southwest China.

This study revealed that infection rates of microsporidia are significantly higher in semi-free red pandas than in captive red pandas (χ2 = 6.212, df = 1, *p* < 0.05). This trend is consistent with an earlier study that has shown a higher prevalence of *Cryptosporidium* spp. in captive and semi-wild orangutans who experienced a higher frequency of human contact compared to wild orangutans [25]. Similarly, Md. Robiul Karim et al. also found that captive non-human primates had a significantly higher prevalence of *E. bieneusi* compared to their free-ranging counterparts [26]. Differences in microsporidia infection rates may be attributed to environmental and host factors, including frequency of contact with humans or other animals, living and health conditions, and other underlying stressors. These findings may indicate that the safety of semi-free roaming behavior needs to be further reconsidered due to the potential for zoonotic transmission. However, due to the incomplete collection of sample information in this study, the age and gender data for certain red pandas could not be obtained, preventing an accurate analysis of the relationship between prevalence and gender and age.

So far, epidemiological investigations of *E. bieneusi* have been carried out on humans, pets, wildlife, and domestic animals [2,27]. The results showed that the total infection rate of *E. bieneusi* in red pandas was 12.1%, which is close to the previous report of red pandas in Shaanxi province, northwestern China (11.1–13.9%) [19]. In this study, the prevalence of *E. bieneusi* in red pandas at Chengdu Zoo and Bifengxia Zoo was 7.1% and 9.1%, respectively, both lower than previously reported rates (10.6% and 29.7%) [20]. Differences in *E. bieneusi* prevalence between studies may be influenced by geographic region, sample size, sampling time, animal health status, activity patterns, and population density [28,29,30]. The raccoon (*Procyon lotor*), as the closest phylogenetic relationship animal with the red panda, had a higher prevalence of *E. bieneusi* (27.3% and 60.0%) [22,31,32]. This may be due to differences in lifestyle or diet, as raccoons are scavengers adapted to survive by consuming a wide range of food, while red pandas primarily feed on bamboo and fruit as herbivores. In addition, the overall prevalence of *E. bieneusi* in red pandas was lower compared to other species commonly found in Chinese zoos with a sampling size of more than 20 (Table 3).

The four genotypes observed in this study were completely different from those previously identified in red pandas in Shaanxi and Sichuan Provinces (QYLP1-3, LGLP1-2, CHB1) [19,20]. PL2, the predominant genotype, was only found in masked palm civets (Paguma larvata) and formed a clade related to Group 2 in the phylogenetic analysis [9]. All positive isolates of *E. bieneusi* from Chengdu Research Base of Giant Panda Breeding were genotyped as PL2, suggesting that the red panda may be a specific host for genotype PL2. The high prevalence of *E. bieneusi* in this location suggests that interspecific transmission of *E. bieneusi* may occur, possibly due to semi-free roaming behavior. However, cross-species transmission could also occur here, as other animals also have a semi-free roaming lifestyle, which increases contact between different species, such as primates and red pandas. Further studies are required to determine whether the semi-free activity pattern increases the potential risk of *E. bieneusi* transmission within and across species in red panda populations. The genotype SC02 has been found in a broad variety of species in China, such as captive giant pandas, pet birds, pet rabbits, and captive black bears [15,41,42,43]. Here, the first SC02 genotype was identified in red pandas. *E. bieneusi* with genotype D was detected in a red panda from both Chengdu Zoo and Bifengxia Zoo. Interestingly, genotype D was also found in other animals in the abovementioned zoos, suggesting the possibility of cross-species transmission of *E. bieneusi* [20]. However, genotype D has been reported to be detected in humans and wild animals, which indicates a potential zoonotic risk [2,44]. The three genotypes (D, SCR1, and SC02) found in the present study belong to group 1 with zoonotic concerns [45]. Although there are no studies showing human infection with animal-derived *E. bieneusi*, close human-animal contact is considered to be a risk factor for microsporidial infection [46]. A study carried out in Peru reported possible transmission between humans and guinea pigs. The same genotype of *E. bieneusi* (Peru16) was identified in a 2-year-old child and guinea pig in the same household [47]. MLST was utilized as a widely accepted genotyping tool to further highlight the genetic variations of *E. bieneusi* in the present study. We found three MLGs (MLG1, MLG2, and MLG3) at the same sampling site, all of which were PL2 genotypes, suggesting that *E. bieneusi* isolates in PL2 genotypes have high genetic diversity.

*Encephalitozoon* spp. was detected in 8 out of the 198 (4.0%) fecal samples from four locations. While the primers used in this study were not specific to *Encephalitozoon* spp., we identified these eight sequences as *Encephalitozoon* spp. based on their high similarity and evolutionary relationships. In this case, specific primers should be used to further identify *E. cuniculi*, *E. hellem* and *E. intestinalis*. Four pairs of primers have been described for the identification of *E. intestinalis*, which amplify the SSU rRNA gene region, including V1/Si500 [48], 3/3 [49], SINTF1/SINTR [50], and V1/Sep1 [51]. The primers ECUNF/ECUNR and EHELF/EHELR were also used for specific identification of *E. cuniculi* and *E. hellem*, respectively [52,53]. In this study, due to the lack of specificity of the primers used for these microsporidian species, we found that the genetic diversity of *E. hellem* and *E. cuniculi* could not be assessed based on the ITS sequences. Therefore, to determine intraspecific variation within *E. hellem* and *E. cuniculi*, another protocol based on the analysis of the polar tube protein gene should be used [54,55]. *Encephalitozoon* spp. has been reported in wild, livestock and companion animals worldwide [56,57,58,59]. This study presents, to the best of our knowledge, the initial data on zoonotic *Encephalitozoon* spp. in red pandas in China. Red pandas may acquire microsporidia in their intestinal tract from various sources such as water, vegetables, and fruits, as prior research has indicated that fresh produce can harbor potentially viable microsporidia spores that pose a risk of infection [60]. Future studies should be conducted to analyze the correlation between microsporidia in food and water sources and microsporidia in the gut of red pandas to further elucidate the potential transmission pathways.

## 5. Conclusions

This study demonstrates the occurrence of *E. bieneusi* infections and *Encephalitozoon* spp. in red pandas. The total infection rate for microsporidia was 15.7%. The semi-free activity pattern may be a potential risk factor for microsporidia infection in red pandas. Four genotypes of *E. bieneusi* were identified, including three known genotypes (SC02, D, and PL2) and one novel genotype (SCR1), with PL2 being the predominant genotype in red pandas. Three MLGs of *E. bieneusi* were identified in red pandas. The results of this study provide data on the occurrence and molecular characteristics of *E. bieneusi* and *Encephalitozoon* spp. in red pandas in Sichuan province, China, and contribute to our knowledge of the genetic diversity and transmission of microsporidia in red pandas.

## Figures and Tables

**Table 1:** Prevalence and genotypic characterization of microsporidia in red pandas in six regions of Sichuan Province. CDZ: Chengdu Zoo; WLCPGSB: Wolong China Panda Garden Shenshuping Base; SLR: Sanlang Resort; BFXZ: Bifengxia Zoo; CDRBGPB: Chengdu Research Base of Giant Panda Breeding; PV: Panda Valley. Group i and group ii represent captive red pandas and semi-free-ranging red pandas, respectively.

Region	Group (i/ii)	No. of Positive/No. of Samples (%)	PCR Positive			
			*E. bieneusi*		*Encephalitozoon* spp.	Mixed Infection
			No. (%)	Genotype (*n*)	No. (%)	No. (%)
CDZ	i	2/11 (18.2)	1 (9.1)	D	1 (9.0)	0
WLCPGSB	i	1/18 (5.6)	0 (0.0)	0	1 (5.6)	0
SLR	i	3/42 (7.1)	3 (7.1)	SCR1	0	0
BFXZ	i	1/14 (7.1)	1 (7.1)	D	0	0
CDRBGPB	ii	22/100 (22.0)	18 (18.0)	PL2	4 (4.0)	0
PV	ii	2/13 (15.4)	1 (7.7)	SC02	2 (15.4)	1 (7.7)
Total		31/198 (15.7)	24 (12.1)	D, SCR1, PL2, SC02	8/198 (4.0)	1 (0.5)

**Table 2:** Multi-locus genotypes of *Enterocytozoon bieneusi* isolated from red pandas in this study. CDRBGPB: Chengdu Research Base of Giant Panda Breeding; SLR: Sanlang Resort; BFXZ: Bifengxia Zoo; PV: Panda Valley.

Sampling Sites	ITS Genotype	Multi-Locus Genotypes				
		MS1	MS3	MS4	MS7	MLGs
CDRBGPB	PL2	-	Type 1	-	Type 1	-
	PL2	-	Type 1	-	Type 1	-
	PL2	Type 1	Type 1	Type 1	Type 1	MLG1
	PL2	Type 1	Type 1	-	-	-
	PL2	-	-	-	Type 1	-
	PL2	Type 1	-	Type 1	Type 1	-
	PL2	-	Type 1	-	-	-
	PL2	-	Type 1	-	Type 1	-
	PL2	-	Type 1	-	Type 1	-
	PL2	Type 1	Type 1	Type 2	Type 1	MLG2
	PL2	Type 1	-	-	Type 1	-
	PL2	Type 1	Type 1	Type 3	Type 1	MLG3
SLR	SCR1	-	Type 1	-	Type 1	-
BFXZ	D	-	Type 2	-	-	-
PV	SC02	-	-	-	Type 1	-

**Table 3:** The prevalence of *Enterocytozoon bieneusi* in other species of Chinese zoos. Only studies with a sampling size of more than 20 were included.

Family	Species	No. of Tested	No. of Positive (%)	Reference
Ailuridae	Red pandas (*Ailurus fulgens*)	198	24 (12.1%)	This study
Cercopithecidae	Golden snub-nosed monkey (*Cercopithecus kandti*)	160	74 (46.2%)	[33]
	Rhesus macaque (*Macaca mulatta)*	411	116 (28.2%)	[34]
	Hamadryas baboon (*Papio hamadryas*)	21	6 (28.6%)	[35]
	Cynomolgus monkey (*Macaca fascicularis*)	62	42 (67.7%)	[26]
Cebidae	Squirrel monkey (*Saimiri* sp.)	43	17 (39.5%)	[35]
	Black-capped capuchin (*Cebus apella*)	22	6 (27.3%)	[35]
Ursidae	Giant panda (*Ailuropoda melanoleuca*)	46	4 (8.7%)	[19]
	Asiatic black bears (*Ursus thibetanus*)	106	29 (27.4%)	[36]
Bovidae	Golden takin *(Budorcas taxicolor bedfordi)*	191	28 (14.7%)	[37]
Anatidae	Whooper swans (*Cygnus cygnus*)	467	35 (7.5%)	[38]
Moschidae	Musk deer (*Moschus berezovskii*)	223	38 (17.0%)	[39]
Lemuridae	Ring-tailed lemur (*Lemur catta*)	45	11 (24.0%)	[35]
Hominidae	Bornean orangutan (*Pongo pygmaeus*)	23	4 (17.4%)	[35]
Macropodidae	Red kangaroo (*Macropus rufus*)	38	14 (36.8%)	[40]

## Data Availability

Nucleotide sequence accession numbers are shown in the article.

## Supplementary Materials

The following supporting information can be downloaded at: https://www.mdpi.com/article/10.3390/ani13111864/s1, Figure S1: Sequence variation in the ITS region between the new SCR1 genotype and the known FJL genotype of *Enterocytozoon bieneusi*; Table S1: All primers, annealing temperatures and amplicon sizes of PCR amplification. References [22,23,24] are cited in the supplementary materials.
