# Supplementary material for: Occurrence and Molecular Characteristics of Microsporidia in Captive Red Pandas (Ailurus fulgens) in China
Source: Animals (Basel). 2023 Jun 3;13(11):1864. doi: 10.3390/ani13111864 (PMC10251819; doi:10.3390/ani13111864)

Supplementary Table S1. all primers, annealing temperatures and amplicon sizes of PCR amplification.

Gene	Primer sequences (5'-3')	AT(℃)	Size(bp)	Reference
E. bieneusi ITS1	F1: GATGGTCATAGGGATGAAGAGCTT	55	-392	[22]
	R1: AATACAGGATCACTTGGATCCGT			
	F2: AGGGATGAAGAGCTTCGGCTCTG			
	R2: AATATCCCTAATACAGGATCACT			
Encephalitozoon spp. ITS	F1: TGAATG(G/T)GTCCCTGT	55	-300	[24]
	R1: TCACTCGCCGCTACT			
	F2: GGAATTCACACCGCCCGTC(A/G)(C/T)TAT			
	R2: CCAAGCTTATGCTTAAGT(C/T)(A/C)AA(A/G)G GGT			
MS1	F1: AAGTTGCAAGTTCAGTGTTTGAA	58	-676	[23]
	R1: GATGAATATGCATCCATTGATGTT			
	F2: TTGTAAATCGACCAAATGTGCTAT			
	R2: ACATAAACCACTAATTAATGTAAC			
MS3	F1: CAAGCACTGTGGTTACTGTT	55	-537	
	R1:AAGTTA GGGCATTTAATAAAATTA			
	F2: GTTCAAGTAATTGATACCAGTCT			
	R2: CTCATTGAATCTAAATGTGTATAA			
MS4	F1: GCATATCGTCTCATAGGAACA	55	-885	
	R1: GTTCATGGTTATTAATTCCAGAA			
	F2: CGA AGTGTACTACATGTCTCT			
	R2: GGACTTTAATAAGTTACCTATAGT			
MS7	F1: GTTGATCGTCCAGATGGAATT	55	-471	
	R1: GACTATCAGTATTACTGATTATAT			
	F2: CAATAGTAAAGGAAGATGGTCA			
	R2: CGTCGCTTTGTTTCATAATCTT			

Supplementary Figure S1. Sequence variation in the ITS region between the new SCR1 genotype and the known FJL genotype of *Enterocytozoon bieneusi*.

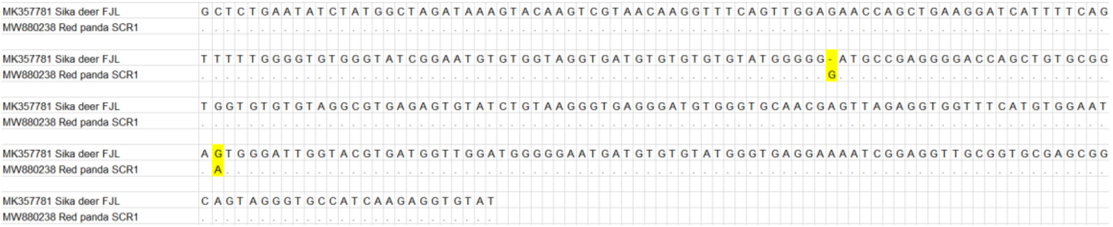

Supplement: Supplementary file 1 [file animals-13-01864-s001.zip › animals-2377547-supplementary.pdf]
